# Patient-Reported Long-Term Gastrointestinal Outcomes in Children with Omphalocele and Gastroschisis: A PedsQL GI Module Study

**DOI:** 10.1055/a-2817-6760

**Published:** 2026-03-12

**Authors:** Maaike Hogerwerf, Adinda G.H. Pijpers, Ramon R. Gorter, Bart G.P. Koot, Ilan J.N. Koppen, Merit M. Tabbers, Joep P.M. Derikx

**Affiliations:** 1 Department of Pediatric Surgery, Amsterdam Pediatric Abdominal Center, Emma Children's Hospital, Amsterdam UMC location University of Amsterdam, Amsterdam, The Netherlands; 2 Department of Pediatric Gastroenterology and Nutrition, Amsterdam Pediatric Abdominal Center, Emma Children's Hospital, Amsterdam UMC location University of Amsterdam, Amsterdam, The Netherlands; 3 Amsterdam Gastroenterology Endocrinology Metabolism Research Institute, Amsterdam UMC Locatie AMC, Amsterdam, The Netherlands; 4 Amsterdam Reproduction and Development Research Institute, Amsterdam UMC Locatie AMC, Amsterdam, The Netherlands

**Keywords:** PedsQL, patient-reported outcome measurements, gastrointestinal, omphalocele, gastroschisis

## Abstract

**Objective:**

Previous studies exploring long-term outcomes of patients with omphalocele and gastroschisis suggest an ongoing burden of gastrointestinal symptoms but also highlight substantial heterogeneity in methodology and outcomes measurement, limiting comparability. Therefore, the aim of this study was to systematically evaluate patient-reported long-term gastrointestinal symptoms, using a validated questionnaire, and compare the scores to those of healthy controls.

**Methods:**

All children treated for omphalocele and gastroschisis between 1999 and 2022 were invited to complete the Pediatric Quality of Life Inventory GI Module, consisting of twelve gastrointestinal domains. Parent proxy-reports or child self-reports were sent by mail, appropriate for the participants' age. The scores were compared with a predefined American healthy cohort ( *n* = 513). Significance threshold was *p* < 0.004 (Bonferroni-corrected).

**Results:**

In total, 45/131 (34.4%) respondents completed the questionnaire, of whom 20 had omphalocele (50.0% male, median age 10.4 years [IQR: 6.4–16.4]), and 25 had gastroschisis (56.0% male, median age 9.9 years [IQR: 6.2–16.8]). Omphalocele patients reported a similar mean total score (86.5 [ ± 15.9] vs. 88.6 [ ± 12.9], *d* = 0.15, *p* = 0.566) and similar domain scores ( *d* < 0.35, *p* > 0.01) compared with healthy controls. Gastroschisis patients also reported a similar mean total score compared with healthy controls (84.9 [SD ± 10.9], *d* = 0.31, *p* = 0.112). However, they scored worse on the domains “gas and bloating” ( *d* = 0.78, *p* = 0.001), “diarrhea” ( *d* = 0.74, *p* = 0.002), and “constipation” ( *d* = 0.51, *p* = 0.026).

**Conclusion:**

Omphalocele patients reported a low burden of long-term gastrointestinal symptoms, indicating that routine follow-up may not be required. In contrast, gastroschisis patients reported a higher prevalence of gas and bloating, diarrhea, and constipation compared with healthy controls, highlighting the importance of early recognition and targeted management of these symptoms.

## Introduction

Omphalocele and gastroschisis are the most common congenital abdominal wall defects in children, with a prevalence of 4 and 2 in 10,000 live births, respectively. 1 Both defects are associated with considerable neonatal morbidity due to surgical challenges and secondary gastrointestinal conditions, such as gastro-esophageal reflux disease, volvulus, necrotizing enterocolitis, or short-bowel syndrome. 2 3 4 5 With improved survival, long-term functioning has become increasingly relevant.

Several studies have explored long-term gastrointestinal outcomes in patients with omphalocele and gastroschisis, yet their findings are heterogeneous with respect to design, assessment tools, and follow-up duration, limiting comparability. 6 7 8 9 10 The only study involving omphalocele patients reported gastrointestinal symptoms in 40% of children, mainly abdominal pain and constipation, with no difference between minor and giant omphalocele. However, these data were collected using a nonvalidated questionnaire and without comparison to healthy controls. 6 For gastroschisis, previous studies have shown gastrointestinal symptoms in up to 75% of patients, most frequently abdominal pain, constipation, and gastro-esophageal reflux disease, which in 30 to 40% of cases even led to hospital readmissions. 7 8 9 Although these studies provided valuable insight, most relied on retrospective chart reviews or nonvalidated questionnaires, and follow-up durations varied widely. More recent studies using the validated Pediatric Quality of Life Inventory (PedsQL) Gastrointestinal Symptoms Scales have provided additional insights. In children with simple gastroschisis, constipation remained the most frequent complaint, though overall gastrointestinal symptom scores were compared with those of healthy controls. 9 In contrast, children with complex gastroschisis reported more symptoms of abdominal pain, gas and bloating, and diarrhea compared with children with simple gastroschisis. 10

Collectively, these findings suggest an ongoing burden of gastrointestinal symptoms in children with omphalocele and gastroschisis but also highlight substantial heterogeneity in methodology and outcomes measurement. This underscores the need for a standardized assessment of long-term functioning to guide parental counseling and follow-up strategies. Therefore, the aim of this study is to evaluate the patient-reported long-term gastrointestinal symptoms of patients with omphalocele and gastroschisis, using the Pediatric Quality of Life Inventory Gastrointestinal Symptoms Module (PedsQL GI Module), a validated tool designed to measure gastrointestinal-specific symptoms and allow for comparison to healthy controls. 11 12

## Methods

### Study Design and Patient Population

All children treated for omphalocele and gastroschisis between 1999 and 2022 in Emma Children's Hospital/Amsterdam University Medical Center, a tertiary center in the Netherlands, were invited to participate in this retrospective cohort study. All patients younger than 2 years of age and older than 25 years of age were excluded from the study.

### Questionnaire

An information letter, informed consent form, and questionnaire with instructions were sent by mail to all (parents of) patients who were eligible. The questionnaire included several questions about the medical and surgical history and the PedsQL GI Module version 3.0, in Dutch. This is a validated and widely used questionnaire. 11 The PedsQL GI Module consists of twelve individual domains, of which ten are Gastrointestinal Symptoms Scales, and two are Gastrointestinal Anxiety Scales. The PedsQL Gastrointestinal Symptoms Scales encompass ten individual domains: abdominal pain; abdominal discomfort when eating; food and drink limits; dysphagia; heartburn and reflux; nausea and vomiting; gas and bloating; constipation; blood in stool; and diarrhea. The questions are answered through a five-point response scale (0 = “never” to 4 = “almost always”). Age-appropriate PedsQL GI Modules were used for each age range. Parent proxy reports were sent to participants aged 2 to 4 and 5 to 7 years. Child self-reports were sent to participants aged 8 to 12 and 13 to 18 years. Adolescent self-reports were sent to participants aged 18 years and older.

### Data Collection

Data collection took place between June 2024 and March 2025. In August 2024 and February 2025, a reminder with a request to participate was sent to eligible participants. The items of the domains of the PedsQL GI Module are reverse-scored and linearly transformed to a 0 to 100 scale (0 = 100, 1 = 75, 2 = 50, 3 = 25, 4 = 0). Lower scores demonstrate more (worse) gastrointestinal symptoms. 13 A total score of every domain was computed as the sum of the items divided by the total number of items for that specific domain. The symptoms' total score of the PedsQL GI Module was computed in a similar manner, as the sum of all items divided by the total number of items in the 10 Symptoms Scales. If > 50% of items in the domain were missing, the domain score was not computed, and the symptom total score was computed using the remaining domains. Moreover, data were extracted from electronic patient records regarding gastrointestinal complaints requiring medical treatment and abdominal complications requiring surgical intervention.

### Statistics

IBM SPSS Statistics for Windows version 28.0 and GraphPad Prism version 10.2.0 were used to perform statistical analyses. Descriptive statistics were presented as frequencies with percentages for categorial data, mean with standard deviation for normally distributed continuous data, or median and interquartile range for nonnormally distributed continuous data. Outcomes of the PedsQL GI Module for patients with omphalocele and gastroschisis were compared with published healthy controls ( *n* = 513) by using an unpaired *t* -test. 12 A Welch's correction was used in case of unequal sample sizes. For this same reason of unequal sample sizes, effect sizes were calculated using the unpooled standard deviation. Due to the published healthy control group, for which only means with standard deviation were available, the scores on the PedsQL GI Module of our study population were also presented and analyzed using means with standard deviation. This approach was applied irrespective of whether the data in our study population followed a normal distribution, to ensure consistency and comparability with the control group. Subgroup analyses were performed for differences between minor versus giant omphalocele, and simple versus complex gastroschisis using Mann–Whitney U tests. To assess potential bias due to a change in reporting perspective, additional subgroup analyses were conducted using Mann–Whitney U tests to identify differences between parent-proxy and child-self reports. Because of multiple testing, the significance level of *p* < 0.05 was adjusted by the Bonferroni correction, after which statistical differences were considered significant if *p* < 0.004.

### Ethical Approval

This study was reviewed by the medical ethical commission and was not subject to the WMO statement (ref. no. W18_233 #18.278, July 2018). Regarding the use of data from the electronic patient dossiers of the Emma Children's Hospital/Amsterdam UMC, written information was provided to (parents or legal guardians of) all identified patients with gastroschisis and omphalocele, including a letter of objection. In case of objection, patients were removed from the database ( *n* = 3). Regarding the use of data from the questionnaire, informed consent was sent to all eligible (parents of) participants. In cases where participants returned the questionnaire but did not provide the signed informed consent, data from the questionnaire were excluded from the study and not used for analysis.

## Results

### Population Characteristics

Between 1999 and 2022, a total of 201 patients with omphalocele and gastroschisis were born at our institute. Of these, eighteen patients had died, and 43 patients were lost to follow-up, leaving 140 patients eligible for inclusion. Nine patients were excluded based on age criteria. Eventually, the questionnaire was sent to 131 patients. A total of 45 (parents of) patients responded to our inventory (34.4%), of which 20 patients had omphalocele and 25 patients had gastroschisis. Ten (50.0%) of the included patients with omphalocele were male, the median age at the time of the survey was 10.4 years (IQR: 6.4–16.4), and fifteen (75.0%) were diagnosed with minor omphalocele. In minor omphalocele, primary surgical closure was the predominant treatment strategy (13/15; 86.7%), with only two patients (13.3%) managed conservatively by allowing epithelialization without secondary surgical closure. For giant omphalocele, four patients (80.0%) were treated by allowing epithelialization, followed by delayed secondary surgical closure using the Ramirez technique, while one patient (20.0%) underwent primary surgical closure. No other treatment strategies, such as staged closure or the use of silo or mesh, were introduced during this study period. Fourteen (56.0%) of the included patients with gastroschisis were male, the median age at the time of the survey was 9.9 years (IQR: 6.2–16.8), and 21 (84.0%) were diagnosed with simple gastroschisis. Seventeen gastroschisis patients (68.0%) underwent primary surgical closure, while eight patients (32.0%) required initial silo placement followed by definitive surgical closure. The healthy control group consisted of a predefined cohort of 513 American children, of whom 46.2% were male, and the mean age was 11.4 years (±4.3). 12 No difference was found in the distribution of sex, median age at survey, and defect severity between responders and nonresponders. Patient characteristics for responders and nonresponders are presented in Table 1 .

**Table 1:** Patient characteristics of responders ( *n* = 45) and nonresponders ( *n* = 86)

		Omphalocele ( *n* = 53)			Gastroschisis ( *n* = 78)		
		Responders ( *n* = 20)	Nonresponders ( *n* = 33)	*p* -Value a	Responders ( *n* = 25)	Nonresponders ( *n* = 53)	*p* -Value a
Sex							
Male		10 (50.0)	13 (39.4)	0.450	14 (56.0)	32 (60.4)	0.714
Female		10 (50.0)	20 (60.3)		11 (44.0)	21 (39.6)	
Age at survey (y)		10.4 (6.4–16.4)	11.2 (5.6–15.6)	0.898	9.9 (6.2–16.8)	12.5 (5.2–18.9)	0.649
PedsQL per age category (y)							
2–4		2 (10.0)			2 (8.0)		
5–7		6 (30.0)			7 (28.0)		
8–12		5 (25.0)			7 (28.0)		
12–17		3 (15.0)			5 (20.0)		
17–25		4 (20.0)			4 (16.0)		
Gestational age (wk)		37.0 (34.3–38.8)			36.0 (35.0–37.0)		
Birthweight (grams)		2,888.3 (2,493.8–3,347.5)			2,500.0 (2,249.0–3,032.5)		
Mode of delivery							
Vaginal		11 (55.0)			17 (68.0)		
Caesarean		9 (45.0)			8 (32.0)		
Type b	Minor	15 (75.0)	22 (66.7)	0.522			
	Giant	5 (25.0)	11 (33.3)				
	Simple	–			21 (84.0)	46 (86.8)	0.741
	Complex	–			4 (16.0)	7 (13.2)	
Associated anomalies		13 (65.0)			6 (24.0)		
Mode of treatment							
Primary closure		14 (70.0)			17 (68.0)		
Silo		–			8 (32.0)		
Epithelization		6 (30.0)			–		

Note: Values are frequencies with percentages (%), mean and standard deviation (SD), or median and interquartile range (IQR), as appropriate.

a Chi-squared test was used to compare the distribution of sex and type of defect between responders and nonresponders. The Mann–Whitney U test was used to compare the median age at survey between responders and nonresponders.

b Gastroschisis was classified as complex when associated with intestinal atresia (jejunal, ileal, or colonic atresia). Omphalocele was classified as giant when the defect diameter was 5 cm or more and/or the omphalocelic sac contained a substantial portion of the liver.

### PedsQL GI Module Scores Omphalocele

The scores of the PedsQL GI Module for patients with omphalocele are presented in Table 2 . Missing data were minimal as only one patient (0.1%) had > 50% missing item responses in two symptom domains and two anxiety domains, for which no domain scores were computed, and no imputation was applied. The mean symptom total score of the PedsQL GI Module of patients with omphalocele was 86.5 ± 15.9 and was not significantly different compared to the healthy controls (88.6 ± 12.9, *d* = −0.15, *p* = 0.566). None of the scores on the individual domains showed a medium or large effect size or differed significantly from the healthy controls. Unfortunately, the small number of patients with giant omphalocele ( *n* = 5) precluded reliable subgroup analyses by complexity.

**Table 2:** PedsQL GI symptoms scales and anxiety scales scores for children with omphalocele and healthy controls

		Omphalocele		Healthy controls			
GI symptoms scales and anxiety scales	Items	*n*	Mean ± SD	*n*	Mean ± SD	Cohen's *d* (unpooled) a	*p* -Value b
Symptoms total score	58	20	86.5 ± 15.9	513	88.6 ± 12.9	−0.15	0.566
Abdominal pain	6	20	80.9 ± 27.9		79.2 ± 19.1	0.07	0.790
Abdominal discomfort when eating	5	20	90.0 ± 16.1		89.6 ± 16.2	0.02	0.914
Food and drink limits	6	20	90.3 ± 16.5		89.7 ± 17.0	0.04	0.875
Dysphagia	3	20	90.4 ± 20.6		95.6 ± 10.9	−0.32	0.275
Heartburn and reflux	4	20	89.1 ± 14.3		90.6 ± 14.3	−0.11	0.650
Nausea and vomiting	4	20	93.1 ± 10.5		91.6 ± 14.7	0.11	0.544
Gas and bloating	7	20	77.7 ± 25.0		83.3 ± 20.1	−0.24	0.334
Constipation	14	19	84.4 ± 22.0		86.9 ± 17.6	−0.12	0.630
Blood in stool	2	20	98.8 ± 3.8		96.3 ± 12.0	0.22	0.017
Diarrhea	7	19	93.0 ± 10.2		94.3 ± 11.5	−0.11	0.593
Anxiety related to defecation	5	19	92.1 ± 23.1		94.2 ± 12.4	−0.09	0.698
Anxiety related to abdominal pain	2	19	85.5 ± 27.7		91.2 ± 16.4	−0.34	0.384

Note: Scores are out of 100. Lower scores demonstrate more gastrointestinal symptoms and lower gastrointestinal-specific HRQOL.

a Effect sizes were calculated using Cohen's *d* with unpooled standard deviation. Effect sizes are designated as small (0.20), medium (0.50), and large (0.80).

b Group differences were analyzed using an unpaired *t* -test with Welch's correction. After Bonferroni correction, *p* < 0.004 is considered significant for group differences.

### PedsQL GI Module Scores Gastroschisis

The scores of the PedsQL GI Module for patients with gastroschisis are presented in Table 3 . Missing data were minimal as only one patient (<0.1%) had > 50% missing item responses in one symptom domain, for which no domain scores were computed, and no imputation was applied. The mean symptom total score of the PedsQL GI Module of patients with gastroschisis was 84.9 ± 10.9 and was not significantly different compared to the healthy controls (88.6 ± 12.9, *d* = −0.31, *p* = 0.112). However, gastroschisis patients scored significantly lower, thus demonstrating more symptoms, on the domains “Gas and bloating” (66.8 ± 22.0 vs. 83.3 ± 20.1, *d* = −0.78, *p* = 0.001) and “Diarrhea” (84.6 ± 14.4 vs. 94.3 ± 11.5, *d* = −0.74, *p* = 0.002), both representing large effects, suggesting moderate to severe symptom burden. Furthermore, medium effects, but not statistically different, were found on the domains “Constipation” (77.4 ± 19.7 vs. 86.9 ± 17.6, d = −0.51, *p* = 0.026) and “Anxiety related to abdominal pain” (80.0 ± 25.0 vs. 91.2 ± 16.4, *d* = −0.53, *p* = 0.036), suggesting moderate symptom burden. Unfortunately, the small number of patients with complex gastroschisis ( *n* = 4) precluded reliable subgroup analyses by complexity.

**Table 3:** PedsQL GI symptoms scales and anxiety scales scores for children with gastroschisis and healthy controls

		Gastroschisis		Healthy controls			
GI symptoms scales and anxiety scales	Items	*n*	Mean ± SD	*n*	Mean ± SD	Cohen's *d* (unpooled) a	*p* -Value b
Symptoms total score	58	25	84.9 ± 10.9	513	88.6 ± 12.9	−0.31	0.112
Abdominal pain	6	25	81.4 ± 18.8		79.2 ± 19.1	0.12	0.573
Abdominal discomfort when eating	5	24	90.4 ± 14.6		89.6 ± 16.2	0.05	0.796
Food and drink limits	6	25	93.8 ± 9.3		89.7 ± 17.0	0.30	0.049
Dysphagia	3	25	97.7 ± 7.0		95.6 ± 10.9	0.27	0.166
Heartburn and reflux	4	25	91.8 ± 12.9		90.6 ± 14.3	0.09	0.655
Nausea and vomiting	4	25	92.1 ± 11.1		91.6 ± 14.7	0.04	0.830
Gas and bloating	7	25	66.8 ± 22.0		83.3 ± 20.1	−0.78	0.001
Constipation	14	25	77.4 ± 19.7		86.9 ± 17.6	−0.51	0.026
Blood in stool	2	25	98.5 ± 5.5		96.3 ± 12.0	0.24	0.080
Diarrhea	7	25	84.6 ± 14.4		94.3 ± 11.5	−0.74	0.002
Anxiety related to defecation	5	25	89.6 ± 14.4		94.2 ± 12.4	−0.34	0.129
Anxiety related to abdominal pain	2	25	80.0 ± 25.0		91.2 ± 16.4	−0.53	0.036

Note: Scores are out of 100. Lower scores demonstrate more gastrointestinal symptoms and lower gastrointestinal-specific HRQOL.

a Effect sizes were calculated using Cohen's *d* with unpooled standard deviation. Effect sizes are designated as small (0.20), medium (0.50), and large (0.80).

b Group differences were analyzed using an unpaired *t* -test with Welch's correction. After Bonferroni correction, *p* < 0.004 is considered significant for group differences.

### Change in Reporting Perspective

As this study included parent-proxy and child-self reports, a supplementary subgroup analysis was conducted to explore whether gastrointestinal symptoms differed between reporting perspectives. Due to the small sample size and to ensure maximum statistical power, the subgroup analysis was performed, including the pooled responses for omphalocele and gastroschisis patients. No significant differences were observed between the symptom total score and domain scores obtained from the parent-proxy reports ( *n* = 18) and child-self reports ( *n* = 27), suggesting that changes in reporting perspectives are unlikely to have influenced the observed outcomes ( Supplementary Table S1 , available in the online version only).

### Abdominal Pathology Requiring Medical Treatment or Surgical Intervention

Table 4 presents gastrointestinal complaints requiring medical treatment and abdominal complications requiring surgical interventions in children with omphalocele and gastroschisis. Five patients (25.0%) with omphalocele experienced gastrointestinal complaints requiring medical treatment at any point after primary discharge, including constipation ( *n* = 3), fecal incontinence ( *n* = 1), feeding difficulties requiring long-term tube feeding ( *n* = 1), and gastroesophageal reflux disease ( *n* = 1). Six patients (30.0%) with omphalocele required surgical interventions for abdominal complications, all within the first 3 years of life (median = 8.0 months, IQR = 2.0–25.8). Two patients developed small bowel obstruction (SBO). One patient with a giant omphalocele treated through epithelialization developed SBO at 2 years of age, requiring adhesiolysis. The other patient, with a minor omphalocele, developed SBO and midgut volvulus without necrosis at 2 months of age, requiring adhesiolysis and derotation of the intestine.

**Table 4:** Gastrointestinal complaints requiring medical treatment and abdominal complications requiring surgical intervention

	Omphalocele ( *n* = 20)	Gastroschisis ( *n* = 25)
	*n* (%)	*n* (%)
Gastrointestinal complaints requiring medical treatment a		
Constipation	3 (15.0)	9 (36.0)
Fecal incontinence	1 (5.0)	0 (0.0)
GERD	1 (5.0)	1 (4.0)
Paralytic ileus	0 (0.0)	2 (8.0)
Short-bowel-syndrome	0 (0.0)	2 (8.0)
Feeding difficulties	1 (5.0)	0 (0.0)
Abdominal complications requiring surgical intervention b		
Small bowel obstruction	2 (10.0)	3 (12.0)
Intestinal perforation	0 (0.0)	1 (4.0)
Intestinal ischemia	0 (0.0)	2 (8.0)
Anastomotic stricture	0 (0.0)	1 (4.0)
Midgut volvulus	1 (5.0)	0 (0.0)
Inguinal hernia	3 (15.0)	1 (4.0)
Umbilical hernia	0 (0.0)	2 (8.0)
Incisional hernia	3 (15.0)	1 (4.0)

Note: Some patients experienced multiple abdominal complaints for which medical treatment or surgical intervention was required.

a At any point after primary discharge.

b At any point after primary surgical closure.

Fourteen patients (56.0%) with gastroschisis experienced gastrointestinal complaints requiring medical treatment, including constipation ( *n* = 9), short-bowel syndrome ( *n* = 2), paralytic ileus ( *n* = 2), and gastroesophageal reflux disease ( *n* = 1). One patient with short-bowel syndrome required total parenteral nutrition (TPN) until the age of 4 years but was fully weaned at the time of this study. The other short-bowel-syndrome patient did not require TPN but received dietary management by a dietitian due to partial malabsorption. Eight patients (32.0%) with gastroschisis required surgical interventions for abdominal complaints at a median age of 4.0 months (IQR = 1.0–46.5). This included ischemia ( *n* = 2) and perforation ( *n* = 1) requiring partial intestinal resection, and anastomotic stricture ( *n* = 1) requiring dilatation, all within the first 4 months of life. Three patients developed SBO. One patient with complex gastroschisis underwent adhesiolysis for SBO at 4 months of age. Two patients with simple gastroschisis each developed two SBO episodes: one at 2 and 3 months of age, requiring adhesiolysis and subsequent partial intestinal resection; the other at 4 and 5 years of age, both treated with adhesiolysis.

## Discussion

This study provides a comprehensive assessment of patient-reported long-term gastrointestinal outcomes in patients with omphalocele and gastroschisis, using the validated PedsQL GI Module and comparison with a predefined healthy cohort. Our findings showed that patients with omphalocele reported a low burden of gastrointestinal symptoms, comparable to healthy controls. Patients with gastroschisis reported a higher prevalence of specific symptoms compared with healthy controls, such as gas and bloating, diarrhea, and constipation.

To our best knowledge, only one other study reported long-term gastrointestinal symptoms in omphalocele patients. Van Eijck et al 6 showed that, with a follow-up of up to 16 years, 40% of patients reported gastrointestinal symptoms as young adults, with 17% experiencing symptoms more than four times a month. The most commonly reported symptoms were abdominal pain and constipation, although no comparison to healthy controls was made. In our cohort, 25% of patients with omphalocele experienced constipation for which medical treatment was required. However, the reported prevalence of constipation in our cohort did not exceed that of healthy controls. The relatively low burden of gastrointestinal symptoms observed in omphalocele patients may be explained by several factors. Unlike gastroschisis, the herniated organs in omphalocele are typically covered by a protective sac, which reduces the risk of a compromised bowel. 14 In addition, primary surgical closure is often feasible, especially in small omphaloceles, and extensive bowel resection is rarely required, preserving bowel function. 3 14

In our cohort, patients with gastroschisis reported a comparable general gastrointestinal symptom burden as healthy controls. However, gas and bloating, diarrhea, and constipation were more prevalent in the gastroschisis group. In 36% of patients, constipation even required medical treatment, suggesting a substantial impact on daily functioning and quality of life. The increased prevalence of these symptoms may be explained by several pathophysiological mechanisms. Gastroschisis is associated with direct exposure of the intestine to amniotic fluid in utero, leading to serositis, bowel wall thickening, and subsequently motility disturbances. 14 Surgical interventions, including resection of compromised bowel and the formation of adhesions, can further disrupt normal gastrointestinal transit and absorption. 15 Additionally, altered gut microbiota and a higher risk of small bowel bacterial overgrowth may contribute to symptoms such as bloating and diarrhea. 16 17

Our findings on gastroschisis patients are consistent with those of De Bie et al, 9 who also used the PedsQL GI Module. Although their study included solely simple gastroschisis patients and had a shorter follow-up duration of 6 years, they also found similar overall gastrointestinal symptoms compared with healthy controls, and worse symptoms of constipation. Other studies focused on hospital admissions as a proxy of symptom severity. Risby et al, 8 with a follow-up of 9 years, showed that 40% of children with gastroschisis reported gastrointestinal symptoms leading to hospital admission, which was significantly higher compared with the 12% hospital admissions in children in the general Danish population. Similarly, Harris et al, 7 with a follow-up of 10 years, reported hospital admission due to abdominal pain in 30% of gastroschisis patients. Unfortunately, the authors did not provide further details about the diagnosis and treatment during hospital admission. The difference in definitions used to report gastrointestinal symptom-burden limits direct comparability. However, the results of these studies all underscore the high prevalence of gastrointestinal symptoms in patients with gastroschisis and emphasize the need for medical evaluation of these symptoms.

In addition to the patient-reported outcomes, surgical abdominal re-interventions, particularly those for SBO, remain a critical outcome measure, as they can significantly impact the clinical course. In our cohort, SBO occurred in 10% of omphalocele patients and 12% of gastroschisis patients, with the oldest patients affected at 2 and 5 years of age, respectively. These numbers are in line with a recent, larger study at our center reporting an incidence of adhesive SBO of 7 and 17% in children younger than 3 years of age with omphalocele and gastroschisis, respectively. 18 Additionally, van Eijck et al 19 showed a 10-year incidence of adhesive SBO of 15 and 37% for omphalocele and gastroschisis patients, respectively.

From a clinical perspective, our findings suggest that routine follow-up regarding gastrointestinal symptoms may not be necessary for omphalocele patients, unless specific symptoms arise. In contrast, for gastroschisis patients, healthcare providers should focus on early recognition and targeted management of gastrointestinal symptoms. Follow-up should include regular symptom-based screening, particularly for constipation, bloating, and diarrhea, ideally during routine surgical or pediatric visits. Patients with persistent or severe symptoms should be referred to a pediatric gastroenterologist for further evaluation and management. Furthermore, given the occurrence of SBO in both omphalocele and gastroschisis groups, parents and patients should be counseled regarding the clinical signs of SBO, with prompt medical evaluation advised if such symptoms occur.

It is important to acknowledge that several potential confounders may influence long-term gastrointestinal outcomes in children with omphalocele and gastroschisis, such as prematurity, nutritional status, and socio-economic background, and should be incorporated in future studies. Future research should also focus on prospective, longitudinal monitoring of gastrointestinal function, ideally combining patients' reported outcomes with clinical data. Research linking symptom burden to surgical factors, such as reoperations or adhesions, as well as exploring the role of neurodevelopment and gut microbiome, may provide further insight into the mechanisms underlying persistent gastrointestinal symptoms in this population.

One of the main strengths of this study is the long follow-up period, with a median age of 10.4 and 9.9 years for omphalocele and gastroschisis patients, respectively. Additionally, we applied a validated questionnaire and compared our results to healthy controls. Notably, the control group was derived from a historical cohort of American children, whereas our study population consisted of Dutch children. 12 This may introduce cultural, demographic, and healthcare-related differences, which could potentially affect score comparisons. The PedsQL GI Module has demonstrated strong psychometric properties in gastrointestinal patient populations, including excellent internal consistency and construct validity. 11 However, formal cross-cultural measurement invariance of this instrument has not been extensively evaluated, and therefore, potential cross-cultural effects on scoring cannot be entirely excluded. Thereby, only means and standard deviations were available for the control group, which precluded full statistical comparison and matching. Moreover, using means and standard deviations without verifying the normality of data may have introduced bias in the presence of skewed data. The use of proxies and self-reported questionnaires could introduce response bias. However, subgroup analyses comparing parent-proxy and child-self reports showed no difference in reported gastrointestinal symptoms. Furthermore, the results are based on patient-reported symptoms, rather than criteria-based diagnoses such as those defined by the Rome IV classification, which could introduce subjectivity and variability in symptom reporting. 20 We have reached a response rate of 34.4%, probably as a result of the long-term follow-up. 21 Although baseline characteristics between the responders and nonresponders were comparable, the possibility remains that selection bias may have affected the reported symptom prevalence. Unfortunately, due to the limited number of respondents, we were unable to conduct subgroup analyses for patients with minor versus giant omphalocele and simple versus complex gastroschisis. Although a substantial proportion of giant omphalocele (25%) and complex gastroschisis (16%) cases were included, the limited sample size in these subgroups may restrict the generalizability of our findings to all patients with larger or more complex defects. These limitations should be considered when interpreting the findings.

## Conclusion

In conclusion, patients with omphalocele reported a low burden of long-term gastrointestinal symptoms, with prevalence rates comparable to those of the healthy controls, indicating that routine follow-up may not be required. In contrast, patients with gastroschisis reported a higher prevalence of gas and bloating, diarrhea, and constipation, highlighting the importance of early recognition and targeted management of these symptoms. These findings can be used to guide parental counseling and inform follow-up strategies for patients with omphalocele and gastroschisis.
