# Supplementary material for: Patient-Reported Long-Term Gastrointestinal Outcomes in Children with Omphalocele and Gastroschisis: A PedsQL GI Module Study
Source: Eur J Pediatr Surg. 2026 Mar 12;36(4):292–9. doi: 10.1055/a-2817-6760 (PMC13375281; doi:10.1055/a-2817-6760)
Supplement: Supplementary file 1 — Supplementary Material [file 10-1055-a-2817-6760_29123626.pdf]

Supplementary Table 1. PedsQL GI Symptoms Scales and Anxiety Scales scores for children with omphalocele and gastroschisis, stratified by parent-proxy and child-self reports.

		**Parent-proxy reports**		**Child-self reports**			
GI Symptoms Scales and Anxiety Scales	*Items*	*n*	*Median (IQR)*	*n*	*Median (IQR)*	*r†*	*p‡*
**Symptoms total score**	**58**	**18**	**92.3 (81.8 - 97.1)**	**27**	**87.9 (73.7 - 94.8)**	0.16	0.276
Abdominal pain	6	18	95.8 (78.6 - 100.0)	27	79.2 (70.8 - 100.0)	0.13	0.367
Abdominal discomfort when eating	5	17	100.0 (87.5 - 100.0)	27	95.0 (80.0 - 100.0)	0.16	0.280
Food and drink limits	6	18	100.0 (93.7 - 100.0)	27	95.8 (87.5 - 100.0)	0.23	0.123
Dysphagia	3	18	100.0 (100.0 - 100.0)	27	100.0 (91.7 - 100.0)	0.12	0.426
Heartburn and reflux	4	18	100.0 (93.8 - 100.0)	27	93.8 (81.3 - 100.0)	0.38	0.011
Nausea and vomiting	4	18	100.0 (93.8 - 100.0)	27	100.0 (81.3 - 100.0)	0.13	0.365
Gas and bloating	7	18	73.2 (56.3 - 97.3)	27	71.4 (46.4 - 89.3)	0.07	0.616
Constipation	14	18	89.3 (70.5 - 97.3)	26	89.3 (67.4 - 96.4)	0.06	0.701
Blood in stool	2	18	100.0 (100.0 - 100.0)	27	100.0 (100.0 - 100.0)	0.25	0.091
Diarrhea	7	18	100.0 (90.2 - 100.0)	26	89.3 (74.1 - 93.8)	0.38	0.012
Anxiety related to defecation	5	18	100.0 (93.8 - 100.0)	26	100.0 (83.8 - 100.0)	0.15	0.328
Anxiety related to abdominal pain	2	18	100.0 (81.3 - 100.0)	26	87.5 (71.9 - 100.0)	0.15	0.327

Note. Parent-proxy reports were sent to children 2 – 7 years of age, and child-self reports were send to children 8 years of age and older.

Scores are out of 100. Lower scores demonstrate more gastrointestinal symptoms and lower gastrointestinal-specific HRQOL.

†Group differences were analyzed using the Mann-Whitney U test. Effect sizes are designated as small (0.10), medium (0.30), and large (0.50).

‡After Bonferroni correction $p < 0.004$ is considered significant for group differences.
